# A Glimpse into Quantum Triplet Structures in Supercritical ^3^He

**DOI:** 10.3390/e25020283

**Published:** 2023-02-02

**Authors:** Luis M. Sesé

**Affiliations:** Departamento de Ciencias y Técnicas Fisicoquímicas, Facultad de Ciencias, Universidad Nacional de Educación a Distancia (UNED), Avda. Esparta s/n, Las Rozas, 28232 Madrid, Spain; msese@ccia.uned.es

**Keywords:** quantum triplet structures, path integral Monte Carlo, closures, supercritical helium-3

## Abstract

A methodological study of triplet structures in quantum matter is presented. The focus is on helium-3 under supercritical conditions (4 < *T*/K < 9; 0.022 < $\rho_{N}/{Å}^{-3}$ < 0.028), for which strong quantum diffraction effects dominate the behavior. Computational results for the triplet instantaneous structures are reported. Path integral Monte Carlo (PIMC) and several closures are utilized to obtain structure information in the real and the Fourier spaces. PIMC involves the fourth-order propagator and the SAPT2 pair interaction potential. The main triplet closures are: AV3, built as the average of the Kirkwood superposition and the Jackson–Feenberg convolution, and the Barrat–Hansen–Pastore variational approach. The results illustrate the main characteristics of the procedures employed by concentrating on the salient equilateral and isosceles features of the computed structures. Finally, the valuable interpretive role of closures in the triplet context is highlighted.

## 1. Introduction

Quantum triplet structure studies are key to developing the statistical mechanics of equilibrium many-body systems at low (nonzero) temperatures. Despite the fact that triplet structures are not determined experimentally [1], the computational approach to this topic not only justifies itself by the gaining of knowledge about quantum matter, but it also is crucial for further applications of quantum reasoning (e.g., phase transitions and design of materials, phonon-phonon interactions in superfluids, time-dependent phenomena, etc.) [2,3,4,5].

As stressed elsewhere [6], exact quantum triplet calculations are presently an extremely demanding task. This contrasts sharply with their counterparts in the classical domain where the calculations are far more affordable [7,8,9,10]. Putting aside the high dimensionality that the triplet functions can reach (e.g., 10-D for spatial triplets in a monatomic solid), one should note the quantum variety of physically significant *n*-particle structures that a system can exhibit in both the real space (**r**-space) and the reciprocal Fourier space (**k**-space) (see [11,12] for a basic description). For the reader to grasp the overall situation, suffice it to consider a monatomic homogenous and isotropic fluid with substantial quantum effects that make a classical description meaningless. This fluid shows six basic triplet functions that can be classified into three types, namely, instantaneous, total continuous linear response, and centroids (the classical counterpart only has two basic functions in a single class) [5,6,11,12]. Each of these three types contains one generalized triplet correlation function $H_{3}\left(r_{12},r_{13},r_{23}\right)$ together with its related Fourier transform $S^{\left(3\right)}\left(k_{1},k_{2},\cos\left(k_{1},k_{2}\right)\right)$, where $r_{jm}=\left|q_{j}-q_{m}\right|$ is the distance between particles *j* and *m*, and $k_{1}$ and $k_{2}$ are two wavevectors of moduli $k_{1}$ and $k_{2},$ respectively. As seen, these triplet functions are already 4-D.

Accordingly, one can obtain an initial impression of the magnitude and expected cost of the related quantum computations, which would increase if higher-order structures were to be dealt with. However, this impression becomes more acute when the inherent features of the actual computations enter the discussion. Very powerful methods to calculate most of the properties of equilibrium quantum condensed matter are based on Feynman’s path integrals (PI) [13]. Within PI two main simulation techniques are available: path integral Monte Carlo (PIMC) and path integral molecular dynamics (PIMD) [11,14,15,16,17,18,19,20,21,22,23,24,25,26,27,28,29,30]. In a way similar to the classical MC and MD techniques, the quantum PIMC and PIMD are highly accurate, their procedural “errors” (e.g., statistical, numerical) being diminished by increasing the simulation run lengths and/or the precision of the computations. By focusing on definiteness for the PIMC simulations of quantum equilibrium structures, they may also be regarded as “exact” in that they provide self-contained solutions unattainable via basic frameworks in statistical mechanics. In relation to this, one should recall the case of the exact (and theoretically revealing) Bogolyubov–Born–Green–Kirkwood–Yvon hierarchy (BBGKY), which needs the knowledge of higher-order structures to define lower-order structures [31]. Thus, although BBGKY is an exact formulation, it leads to calculational schemes (not in use) that cannot be “exact”, since they need extra information to break/close the working equations (e.g., hypothesis about the form of triplet correlations -*closures*- to calculate the pair correlations). This does not occur with PIMC, which is self-contained (e.g., pair and triplet correlation functions can be calculated independently of each other) and whose accuracy can in principle be arbitrarily increased so as to reproduce the targeted theoretical values of the model selected [14,15,18]. Note that the foregoing polysemous use of the term “exact” is independent of another use referring to the computational effort required to deal with an increasing number of particles in a system.

The PI difficulty lies in the extended simulation samples $N_{S}\times P$ that PIMC and PIMD use; $N_{S}$ stands for the conventional number of actual particles, and P>1 is an integer number to be optimized that serves to represent the thermal quantum delocalization of an actual particle (theoretical accuracy is reached in the Trotter’s limit P→∞) [18]. As a rule, *P* increases with the quantum effects and there are ways to soften its impact on the number of calculations (e.g., pair actions [18,19], fourth-order propagators [20,25,26,27,28,29,30], parallel computing [20], etc.). All in all, when very strong quantum effects (including bosonic exchange [18,23,28]) are to be studied, the PI undertaking of the triplet task in its entirety, and within reasonable CPU times (and electric power consumption), may remain today out of the reach of most interested researchers. In this regard, note that triplet **r**-space information, though expensive, is still affordable, whereas its counterpart in **k**-space is highly demanding because of the necessity to scan appropriate sets of **k** wavevectors commensurate with the simulation box. It thus seems that the related numerical evaluations of detailed quantum **r**- and **k**- structures for triplets (and beyond) could be appealing targets for the PI implementations in the coming exascale computers [32]. For completeness, note that, apart from the present author’s works, PI work on significant aspects of the equilibrium structures in quantum matter can also be found in the general references [14,15,16,17,18,19,20,21,22,23,24,25,26,27,28,29,30], more specifically [14,16,18,20,27,28,29,30]. However, none of the latter deals with all the aforementioned types, nor goes beyond the pair level.

For the time being, the main structural features of triplets in fluids with quantum behavior can be extracted by means of PIMC (or PIMD). Recent works by the present author have shown how to tackle this problem when dealing with quantum diffraction effects [2,6,33,34,35,36]. In these works, one can find comprehensive descriptions of the methods employed, and also positive identifications (or well-grounded indicators) of physically significant triplet patterns in the statistical distributions of the actual particles. The systems studied were the quantum hard-sphere fluid, bare [2,33] and with Yukawa attractions [35], helium-3 at very low densities [34], liquid neon [6], and liquid para-hydrogen [6,36]. By defining the parameter $\gamma =\rho_{N}\lambda_{B}^{3}$ as a (rough) measure of the magnitude of the quantum effects, where $\rho_{N}$ is the number density, and $\lambda_{B}=h/\sqrt{2\pi mk_{B}T}$ is the thermal de Broglie wavelength, the studied conditions covered up to γ≲2.7.

As complementary tools, one can also employ the so-called closures, which for triplets intend to infer their characteristics from the available information at the pair level: $g_{2}\left(r\right)$ correlations, $c_{2}\left(r\right)$ direct correlation functions, $S^{\left(2\right)}\left(k\right)$ structure factors, and other auxiliary functions [3,5,37]. Closures are certainly approximations and imply far less expensive calculations than the exact PI techniques. Furthermore, closures may turn out to be highly accurate, as shown at the pair level [11,38], or, alternatively, very useful as interpretive tools for analyzing complex structural problems [2,6,36]. From the point of view of the present author, any theoretical object allowing further reasoning and a deeper understanding always deserves careful study, and this turns out to be the case of closures in the quantum domain.

In the hope of shedding some more light on quantum triplet structures, and as a preliminary part of a larger project, this article addresses some relevant issues. The focus is on the instantaneous triplet structures in the **r** and the **k** spaces of supercritical helium-3. The triplet behavior is explored for conditions $\left(4<T\left(K\right)<9;0.022<\rho_{N}\left({Å}^{-3}\right)<0.028\right)$ (critical point: $T_{C}=3.3157K;\rho_{N}=41.191\frac{kg}{m^{3}}≅$ 0.0082246 ${Å}^{-3}$) [39]. Why helium-3 under these conditions? One obvious reason is to give a service by communicating more experience on the, as yet, unexplored triplet topic. Another reason is that quantum diffraction effects (γ≲3.2) beyond those mentioned above can be analyzed in (a model of) a real system as important as helium-3. Furthermore, the rationalization of the quantum triplet structures in terms of closures built from the underlying pair structures is worth pursuing [2,6,36]. The supercritical conditions are far from fermionic exchange, known to be present for T≤1K and affected by the computational “sign problem”, which precludes practical applications of PIMC (see [17] for a pioneering PIMC approach to this problem). In this article, the equilateral and isosceles correlations are determined in **r**-space with PIMC and closures, and in **k**-space with closures. PIMC involves the fourth-order propagator put forward in [25,26,27] (compare with the early application of the primitive propagator that was utilized in [34] for the study of helium-3 at very low densities). The triplet closures employed are Jackson–Feenberg convolution JF3 [3], Kirkwood superposition KS3 [37], the intermediate AV3 = (KS3 + JF3)/2 [2], and the variational Barrat–Hansen–Pastore approach (BHP) [5]. KS3, JF3, and AV3 are utilized for **r**-space and **k**-space, whereas BHP is utilized only for **k**-space. The effects in **r**-space arising from changes in temperature and in density are discussed, and the significant role of the closures is highlighted.

The outline of this article is as follows. Section 2 contains a summary description of the underlying theory and methods. Section 3 is devoted to the main computational details, and Section 4 gives the results and their discussion. Finally, Section 5 collates the conclusions of this work.

## 2. Theory

### 2.1. Path Integral Monte Carlo

For a monatomic homogeneous and isotropic fluid at equilibrium, in which quantum exchange can be neglected, the general form of the PI-canonical (N,V,T) partition function reads as [14,18]

(1) $$Z_{NP}={\left(N!\right)}^{-1}{\left(\frac{mP}{2\pi \beta \hbar^{2}}\right)}^{3NP/2}\int \prod_{t=1}^{P}dr^{N,t}exp\left[-\beta W_{NP}\left(r^{N,1},\ldots ,r^{N,t};\beta ,\hbar ,m\right)\right]$$

where *m* is the particle mass, $\beta =\frac{1}{k_{B}T}$ is the inverse temperature, the actual *N* particles (j=1,2,…,N) are represented by *N* necklaces with *P* beads apiece (t=1,2,…,P), $r^{N,t}=\left(r_{1}^{t},\ldots ,r_{N}^{t}\right)$ where $r_{j}^{t}$ denotes the coordinates of bead *t* belonging to necklace *j*, $dr^{N,t}=dr_{1}^{t}\ldots dr_{N}^{t},$ and $W_{NP}$ is the effective potential for the whole set of N×P beads. Equation (1) resembles the form of a semiclassical partition function [11,13], which gives rise to the so-called (semi-)classical isomorphism [14] that is at the root of the great PI success. The number *P* is assumed to be optimized in what follows.

The specific expression of $W_{NP}$ depends on the selection of the thermal propagator. In this work this propagator is the fourth-order choice in the final form given by Voth et al. to the Suzuki-Chin developments [25,26,27]. In this case *P* is an even integer, and *W_NP_* is given by

(2) $$W_{NP}=\frac{mP}{2\beta^{2}\hbar^{2}}\overset{N}{\underset{j=1}{\sum }}\overset{P*}{\underset{t=1}{\sum }}{\left(r_{j}^{t}-r_{j}^{t+1}\right)}^{2}+\frac{2}{3P}\underset{j<m}{\sum }\left\{\underset{t=odd}{\sum }v\left(r_{jm}^{t}\right)+2\underset{t=even}{\sum }v\left(r_{jm}^{t}\right)\right\}+\;\frac{\beta^{2}\hbar^{2}}{9mP^{3}}\sum_{j=1}^{N}\left\{\alpha \sum_{t=odd}{\left(\sum_{j\neq m}\frac{dv\left(r_{jm}^{t}\right)}{dr_{jm}^{t}}\eta_{jm}^{t}\right)}^{2}+\left(1-\alpha \right)\sum_{t=even}{\left(\sum_{j\neq m}\frac{dv\left(r_{jm}^{t}\right)}{dr_{jm}^{t}}\eta_{jm}^{t}\right)}^{2}\right\},$$

where the * in the first *t*-summation implies the cyclic property t+1=P+1≡1, α is a real number in the interval [0, 1], $v\left(r_{jm}^{t}\right)$ is the continuous pair potential v(r) acting between equal-*t* beads in different necklaces, $r_{jm}^{t}=\left|r_{j}^{t}-r_{m}^{t}\right|,$ $\eta_{jm}^{t}=r_{jm}^{t}/r_{jm}^{t},$ and the asymmetry in the bead contributions coming from odd-*t* (t=1,3,…,P−1) and even-*t* (t=2, 4,…,P) is to be noticed. This asymmetry has an obvious impact on the thermodynamic evaluations, but it is of critical importance to the structural computations, for only the odd-numbered beads are physically significant. The reader is referred to [11,27,28] for the related discussions.

By focusing only on the instantaneous structures (ET for equal-*t*) at the fluid number density $\rho_{N}=N/V$, one finds at the pair and triplet levels the following direct canonical averages ⟨…⟩ in the **r**- and **k**- spaces

(3) $$\rho_{N}^{2}g_{ET2}\left(q_{1},q_{2}\right)=\rho_{N}^{2}g_{ET2}\left(r_{12}\right)=\frac{2}{P}\langle \sum_{j\neq m}\sum_{t=1,3,\ldots ,\;P-1}\delta \left(r_{j}^{t}-q_{1}\right)\delta \left(r_{m}^{t}-q_{2}\right)\rangle ,$$

$$\rho_{N}^{3}g_{ET3}\left(q_{1},q_{2},q_{3}\right)=\rho_{N}^{3}g_{ET3}\left(r_{12},r_{13},r_{23}\right)=$$

(4) $$\frac{2}{P}\langle \sum_{j\neq m\neq n\neq j}\sum_{t=,1,3,\ldots ,\;P-1}\delta \left(r_{j}^{t}-q_{1}\right)\delta \left(r_{m}^{t}-q_{2}\right)\delta (r_{n}^{t}-q_{3})\rangle ,$$

(5) $$S_{ET}^{\left(2\right)}\left(k\right)=\frac{2}{NP}\langle \sum_{j=1}^{N}\sum_{m=1}^{N}\sum_{t=1,3,\ldots ,P-1}exp\left[i\;k\cdot \left(r_{j}^{t}-r_{m}^{t}\right)\right]\rangle_{,}$$

$$S_{ET}^{\left(3\right)}\left(k_{1},k_{2},\cos\gamma \right)=$$

(6) $$\frac{2}{NP}\langle \sum_{j=1}^{N}\sum_{m=1}^{N}\sum_{n=1}^{N}\sum_{t=1,3,\ldots ,P-1}exp\left[i\left(k_{1}\cdot r_{j}^{t}+k_{2}\cdot r_{m}^{t}-\left(k_{1}+k_{2}\right)\cdot r_{n}^{t}\right)]\right\rangle .$$

In connection with the foregoing formulas, some remarks are to be made: (a) $q_{j}$ stands for the absolute position vector of the actual particle *j*, so that $r_{jm}=\left|q_{j}-q_{m}\right|$ is the distance between the actual particles *j* and *m*; (b) in Equations (5) and (6) terms of the δ(k)− type are omitted, as is customary [40,41]; (c) γ is the angle between the wavevectors $k_{1}$ and $\;k_{2};$ and (d) while $S_{ET}^{\left(2\right)}\left(k\right)$ is essentially the Fourier transform of $h_{ET2}\left(r_{12}\right)=g_{ET2}\left(r_{12}\right)-1,\;S_{ET}^{\left(3\right)}$ is the Fourier transform of an involved **r**-space function, $H_{ET3}\left(r_{12},r_{13},r_{23}\right)$ **[6,11]**. The latter not only contains $g_{ET3}\left(r_{12},r_{13},r_{23}\right)$, but also additional terms which include two- and one-body contributions. It is worth stressing that Equations (3)–(6) are the common quantities in the structural study of fluids at equilibrium. Actually, $S_{ET}^{\left(2\right)}\left(k\right)$ is directly related to the system response that can be obtained in X-ray or elastic neutron diffraction experiments [18,31]. However, for many purposes, the grand canonical extensions are essential, as will be considered later on (see [6,11] for the other functions and their physical meanings).

There are well-known problems with the simulation of Equations (5) and (6) in the low-*k* regions, caused by the finite particle sample size $N_{S}$ (or volume $V_{S}$) and the required commensurability of the wavevectors with the central box [40,41]. For a cubic box of length *L* the allowed wavevectors must be of the form $k=\left(\frac{2\pi }{L}\right)\left(k_{x},k_{y},k_{z}\right)$ with the $k_{x},\;k_{y},\;k_{z}$ components being integer numbers, and a summary description of these problems seems worth including here (see [11] for a review and complete discussion).

First, for example, one may focus just on the pair level for simplicity and also for its immediate repercussions. Note that the central thermodynamic connection $S_{ET}^{\left(2\right)}\left(k=0\right)=\rho_{N}k_{B}T\chi_{T}$ cannot be strictly determined via a single direct simulation $(\chi_{T}=$ isothermal compressibility), since *k* and $N_{S}$ extrapolations should be carried out [41]. (Due to the lack of a δ(k)-term, the case k=0 in Equation (5) yields an expression far from the true physical meaning of this component [11,42]). Further, the long-range oscillations about zero of $h_{ET2}\left(r_{12}\right)$ cannot be determined with reasonably workable finite sample sizes (unless one had access to computational resources whose use could be hardly justifiable). Although, these oscillations are small in magnitude, they are *decisive* to obtain a significant value of $S_{ET}^{\left(2\right)}\left(k=0\right)$ via the Fourier transform of $h_{ET2}\left(r_{12}\right).$ Nothing of this is new, as this sort of problem was already known in classical statistical mechanics, and was *circumvented* via the use of the Ornstein–Zernike pair scheme (OZ2) with the introduction of the short-ranged direct correlation function $c\left(r_{12}\right)$ [40,41]. One might argue that the finite sample size affects the calculation of every property in a simulation and that there is nothing about the $S_{ET}^{\left(2\right)}\left(k=0\right)$ evaluation, via the volume integral of $h_{ET2}\left(r_{12}\right),$ that is not shared by other properties, such as the energy or the pressure, which can also be formulated with the use of integrals involving the pair function $g_{ET2}\left(r_{12}\right)$ (and the interparticle potential) [18,41]. However, it is worth recalling that, when evaluating energies or pressures of systems composed of electrically neutral particles, the standard way to cope with the finite size effects is to correct the simulation results with continuum contributions. The latter can be evaluated by defining the pair function involved as $g_{2}\left(r_{12}\right)=1$ for distances longer than half the box length L/2. These corrections account for the missing long-range interactions, thereby being fully dependent on the features of the interparticle potential [41], but not on the complete structure of the actual system studied. As stated above, and apart from well-known asymptotic behavior questions [42], the latter definition, $g_{2}\left(r_{12}\right)=1$, would be meaningless when Fourier transforming $h_{2}\left(r_{12}\right)$, and the corresponding argument is flawed.

Second, the sampling of commensurate wavevectors implies the definitions of sets ${\left\{k_{uv}\right\}}_{v=1,2,3,\ldots }$ compatible with the wavenumbers $k_{u}=\left|k_{uv}\right|$ to be analyzed (as many sets as wavenumbers). Therefore, and focusing on the use of (6) for triplets, the increased computational cost involved prompts one to look for reliable alternatives. In this regard, there are methods [11] based on the use of Ornstein–Zernike (OZn) schemes [43] that, while in waiting for a widespread availability of exascale computers, can be explored in the quantum domain for dealing with these serious triplet drawbacks. The OZn schemes are deeply rooted in the grand canonical ensemble developments in both the classical [43] and the quantum [11,14] domains and utilize the aforementioned closures. They are known to produce highly accurate quantum frameworks at the pair level for the three types of pair $S^{\left(2\right)}\left(k\right)$ [12,38] (for PI centroids the chain of OZn schemes is exact [34]). Now, before entering the discussion on closures, three outstanding aspects of the quantum OZ2 treatments deserve to be mentioned. First, the high accuracy obtainable over the whole range of wavenumbers k≥0, even with the use of moderately sized PIMC samples that provide the basic pair structures $g_{2}\left(r\right).$ Second, the excellent agreement with experiment that they produce, and third, their cost-effective character, since they only require a very low computational cost as compared to PI simulations of the structure factors. For more information on these important conceptual and practical subjects, the reader is referred to [2,6,11,33,34,35,36,38].

### 2.2. Closure Approximations

The closures in **r**-space for the instantaneous triplet correlations employed in this work are the following: Kirkwood superposition (KS3) [37], Jackson–Feenberg convolution (JF3) [3], and their average AV3=(KS3+JF3)/2 [2]. By making use of the following conventions: $g_{ET2}\left(r_{jm}\right)\to g\left(r_{jm}\right)$, and $h_{ET2}\left(r_{jm}\right)=g_{ET2}\left(r_{jm}\right)-1\to h\left(r_{jm}\right)=g\left(r_{jm}\right)-1,$ these closures can be cast as

(7) $$g_{ET3}^{KS3}\left(r_{12},r_{13},r_{23}\right)=g\left(r_{12}\right)g\left(r_{13}\right)g\left(r_{23}\right),$$

(8) $$g_{ET3}^{JF3}\left(r_{12},r_{13},r_{23}\right)=g_{ET3}^{KS3}\left(r_{12},r_{13},r_{23}\right)-h\left(r_{12}\right)h\left(r_{13}\right)h\left(r_{23}\right)+\rho_{N}\int dq_{4}h\left(r_{14}\right)h\left(r_{24}\right)h\left(r_{34}\right),$$

(9) $$g_{ET3}^{AV3}\left(r_{12},r_{13},r_{23}\right)=\frac{\left(g_{ET3}^{KS3}\left(r_{12},r_{13},r_{23}\right)+g_{ET3}^{JF3}\left(r_{12},r_{13},r_{23}\right)\right)}{2}=\;1+h\left(r_{12}\right)+h\left(r_{13}\right)+h\left(r_{23}\right)+h\left(r_{12}\right)h\left(r_{23}\right)+h\left(r_{13}\right)h\left(r_{23}\right)+h\left(r_{12}\right)h\left(r_{13}\right)+\;\frac{1}{2}\;h\left(r_{12}\right)h\left(r_{13}\right)h\left(r_{23}\right)+\frac{\rho_{N}}{2}\int dq_{4}h\left(r_{14}\right)h\left(r_{24}\right)h\left(r_{34}\right).$$

Equation (9) shows the form of a truncated *h*-expansion in which the triple *h*-product, subtracted from JF3, is recovered. Surprisingly, this form is more useful than expected, as shown in a recent study of the quantum hard-sphere fluid along the crystallization line [2].

The closures in **k**-space for the instantaneous calculations contained in this work are based on the OZ direct correlation functions $c_{ET2}\left(r_{12}\right)$ and $c_{ET3}\left(r_{12},r_{13},r_{23}\right)$, together with their corresponding Fourier transforms $c_{ET}^{\left(2\right)}\left(k\right)$ and $c_{ET}^{\left(3\right)}\left(k_{1},k_{2},\gamma \right)$ [11]. These schemes lead to the following classical-like recipes for the pair and triplet structure factors

(10) $$S_{ET}^{\left(2\right)}\left(k\right)=S_{ET}^{\left(2\right)}\left(k\right)\approx {\left(1-\rho_{N}c_{ET}^{\left(2\right)}\left(k\right)\;\right)}^{-1},$$

(11) $$\;S_{ET}^{\left(3\right)}\left(k_{1},k_{2}\right)=S_{ET}^{\left(3\right)}\left(k_{1},k_{2},\gamma \right)\approx S_{ET}^{\left(2\right)}\left(k_{1}\right)S_{ET}^{\left(2\right)}\left(k_{2}\right)S_{ET}^{\left(2\right)}\left(\left|k_{1}+k_{2}\right|\right)\{1+\rho_{N}^{2}c_{ET}^{\left(3\right)}\left(k_{1},k_{2}\right)\}.$$

Equations (10) and (11) are approximations to the instantaneous case within a grand canonical ensemble description. However, the pair level Equation (10) is highly accurate for fluids with substantial quantum behavior [11]. Moreover, the latter assertion is true for the fluid phases of helium in which quantum exchange is negligible. This was demonstrated for ^3^He and ^4^He at 4.2K in [38], where one can observe the almost *indistinguishability* between the OZ2 and the exact PIMC results. It is worth remarking that the calculations in **r**-space and in **k**-space can be interconnected through closures, which allows one to achieve excellent approximations to the grand canonical ensemble structures without performing simulations in this ensemble. Thus, by starting from the PIMC canonical pair structures $g_{ET2}\left(r_{12}\right)$ and subjecting them to an extended OZ2 treatment, one can obtain a set of improved functions, $g_{ET2}\left(r_{12}\right)$, $c_{ET2}\left(r_{12}\right)$, and $S_{ET}^{\left(2\right)}\left(k\right),$ which incorporate grand canonical corrections. The latter contribute to correct the deficient asymptotic behavior of the canonical pair radial correlations [42]. Hereafter, this method will be referred to as BDH+BHw (Baxter–Dixon–Hutchison procedure plus Baumketner–Hiwatari corrections) [44,45,46], of which there is plenty of information regarding quantum applications (see [11] and references therein). The calculation of $S_{ET}^{\left(2\right)}\left(k=0\right)$ is just a by-product that arises directly from this approach and provides very valuable estimates of the isothermal compressibility. (As a matter of fact, the exact value of the latter quantity for the model system under study can be obtained via application of OZ2 to the centroid functions [12]). In addition, the complete knowledge of $S_{ET}^{\left(2\right)}\left(k\right)$ allows one to extend, through the inverse Fourier transform, the pair radial correlations $g_{ET2}\left(r_{12}\right)$ up to arbitrarily long distances. Clearly, the latter operation benefits the closure calculations of triplets [11]. Furthermore, the knowledge of $c_{ET2}\left(r_{12}\right)$ over a range of densities, at constant temperature, is key to tackling the far more intricate problem posed by $c_{ET}^{\left(3\right)}\left(k_{1},k_{2},\gamma \right)$.

The calculation of $c_{ET}^{\left(3\right)}\left(k_{1},k_{2},\gamma \right)$ depends heavily on the closure selected [5,10,33,34,35,36]. Apart from the “trivial” JF3 solution $c_{ET}^{\left(3\right)}=0$, which makes JF3 a compulsory reference, and the KS3 solution [5], the present work makes use of the elaborate variational procedure proposed by Barrat, Hansen, and Pastore (BHP) in the classical domain [5]. Therefore, the calculations below are based on the following equations

(12a) $$\frac{\partial c_{2}\left(r_{12};\rho_{N}\right)}{\partial \rho_{N}}=\int dq_{3}\;c_{3}\left(r_{12},r_{13},r_{23}\right),T=constant,$$

(12b) $$c_{3}\left(q_{1},q_{2},q_{3}\right)=c_{3}\left(r_{12},r_{13},r_{23}\right)\approx t\left(r_{12}\right)t\left(r_{13}\right)t\left(r_{23}\right),$$

(12c) $$ℑ\left[t\left(r\right)\right]=4\pi \int_{0}^{\tilde{R_{max}}}dr\;r^{2}{\left\{\frac{\partial c_{2}\left(r;\rho_{N}\right)}{\partial \rho_{N}}-t\left(r\right)\int_{0}^{\tilde{R_{max}}}ds\;t\left(s\right)t\left(\left|r-s\right|\right)\right\}}^{2}$$

For simplicity of notation, the dependence of the direct correlation functions on $\rho_{N}$ is included only in $c_{2}\left(r\right)$. The auxiliary function t(r) defines the BHP closure by cutting Baxter’s $c_{n}$-hierarchy [47] at the triplet level, Equation (12a). The fixing of t(r) is performed through the functional minimization of ℑ[t(r)] $(\tilde{R_{max}}\;$ is an upper limit for the integrations in Equation (12c)). Once t(r) is obtained, the double Fourier transform of $c_{3}$ leads to $S^{\left(3\right)}\left(k_{1},k_{2}\right)$ [5]. Also, note the exact relationship arising from Equation (12a)

(12d) $$\frac{\partial c^{\left(2\right)}\left(k_{1}\right)}{\partial \rho_{N}}=c^{\left(3\right)}\left(k_{1},k_{2}=0\right),\;\;\;T=constant,$$

The BHP applications to the ET2 case make the replacements of the pair and triplet (BHP classical) quantities by their quantum instantaneous analogs. By taking advantage of Equation (12d), approximate estimates of the double-zero momentum transfer $S_{ET}^{\left(3\right)}\left(k_{1}=0,k_{2}=0\right)$ can be obtained. (In actual fact, the use of the exact centroid function framework would yield the exact value of the latter component) [6,34]. Although Equation (12b) is already an approximation to the exact classical behavior of $c_{3},$ BHP is known to capture some interesting traits of quantum triplet structure factors [36]. Whether or not BHP may yield quantum triplet results of a quality comparable to that achievable at the pair level with BDH+BHw, only further applications and comparison with PI results will settle the question. Therefore, BHP is utilized in this work for continuing the pilot exploration of such topic when strong quantum diffraction effects are involved.

## 3. Computational Details

The helium-3 state points studied are: SP1 $(T=4.21\;K;V=26.33\frac{cc}{mol};\rho_{N}≅0.022872\;{Å}^{-3}$), SP2 $(T=4.21\;K;V=22.06\frac{cc}{mol};\rho_{N}≅0.027299\;{Å}^{-3}$), SP3$\left(T=8.99\;K;V=26.33\frac{cc}{mol};\;\rho_{N}≅0.022872\;{Å}^{-3}\right),$ and SP4 $(T=4.2\;K;\;V=26.3353412\frac{cc}{mol};\;\rho_{N}≅0.022867\;{Å}^{-3}$). The atom mass of helium-3 is set to 3.01603 amu. Conditions SP1 to SP3 are taken from [48], allowing the comparison of the **r**-space triplet instantaneous results under independent variations of temperature and density. The selection of SP4 is made to carry out the triplet instantaneous calculations in **k**-space with closures. SP4 and its adjacent states along the 4.2 K isotherm were studied in [38], where their $c_{ET2}\left(r_{12}\right)$, $c_{ET}^{\left(2\right)}\left(k\right),$ and $S_{ET}^{\left(2\right)}\left(k\right)$ functions were obtained via BDH+BHw. For purposes of interpretation of the results, the SP1-SP4 closeness is an advantage worth exploiting in this work.

The PIMC simulations follow the general lines already described in other works by the present author [33,38], and only a summary is given here. The interatomic potential employed is SAPT2 [49,50], which produces very reliable results for this system [11,38]. Consequently, SAPT2 can be regarded as adequate for the present structural purposes. The canonical ensemble is used for the basic **r**-space calculations, involving the fourth-order propagator $\left(\alpha =\frac{1}{3}\right)$ [27], and with the sample sizes: $N_{S}\times P=$ 1372×66 (SP1), 1372×80 (SP2), and 1372×22 (SP3) (state point SP4 was studied with 1024×66 in [38]). The necklace normal-mode algorithm [51] is utilized, and the usual Metropolis sampling procedure is applied by setting the acceptance ratio for the different *P*-moves to 50%. One kpass is defined as $10^{3}N_{S}\times P$ attempted bead moves. As stated earlier, the canonical pair instantaneous structures $g_{ET2}\left(r\right)$ are needed to undertake the calculations of the triplet instantaneous closures, and the run lengths to obtain these $g_{ET2}\left(r\right)$ are in between 500 kpasses and 2000 kpasses. The triplet instantaneous structures computed, $g_{ET3}\left(r,s,s\right),$ are fixed with run lengths in between 750 and 3660 kpasses. The sampling of the pair and triplet structures uses a spacing in the interparticle distances set to $\Delta_{g}=0.1\;Å$ [33]. The statistical error bars remain controlled: for example, at the first peaks of $g_{ET2}\left(r\right)$ one finds the error bars (one standard deviation) well below 1%, and at the first peak of the equilateral $g_{ET3}\left(r,r,r\right)$ one finds that the error bars remain ≤1% (see the Supplementary Material). The current applications using the PI fourth-order propagator employed [27] should be compared to those of the primitive propagator reported more than a decade ago in [34], where gaseous helium-3 was studied at 5.23 K and very low densities $\left(<0.0021\;{Å}^{-3}\right)$ with sample sizes having: $N_{S}=108,500,P\leq 130$. The reduction in *P* and the possibilities for increasing $N_{S}$ (or equivalently, for analyzing wider ranges of the **r**-correlations) are powerful advantages offered by this efficient propagator when studying increasing densities.

Real space triplet calculations with closures use as data input the improved PIMC-$g_{ET2}\left(r\right)$ functions, which are extended up to distances longer than half the box-length L/2 with the use of $S_{ET}^{\left(2\right)}\left(k\right).$ At a given state point, after calculating the PIMC-$g_{ET2}\left(r\right),$ this task is accomplished in three steps: (1) application of Baxter–Dixon–Hutchinson’s treatment (BDH) of the OZ2 equation [44,45]; (2) fixing of grand canonical ensemble corrections (BHw: five iterations) [46]; and (3) Fourier inversion of $S_{ET}^{\left(2\right)}\left(k\right).$ (For details see [11,38,52,53]). The crucial point is that $c_{ET2}\left(r\right)$, which is short ranged, is fixed over a finite range $R_{Z}$ of distances: $c_{ET2}\left(r\geq R_{Z}\right)=0$. In this regard, there may appear more than one $R_{Z}$ value (hereafter $R_{Z}$-zeros), for which the main part of $c_{ET2}$ is kept essentially invariable, but obviously they yield different tails for the $c_{ET2}$ decay towards zero with increasing *r* [53]. These tails only have (generally) a small effect on $S_{ET}^{\left(2\right)}\left(k\right)$ in the region of very low-*k* values (see [38] for noticeable exceptions), and an effective averaging method has been proposed to deal with this situation [53,54]. However, this tail effect may or may not become important when the density derivatives involving isothermal sets $\left\{c_{ET2}\left(r;\rho_{N};R_{Z}\right)\right\}$ must be computed, and the results below illustrate this point. Also, closure results for KS3 Equation (7) are trivial, but those for JF3 and AV3 depend on the convolution integral shown in Equation (8), which contains the total correlation function $h\left(r\right)\equiv h_{ET2}\left(r\right)$. It is worthwhile to mention that, in evaluating this convolution, use of a well-known expansion in Legendre polynomials $P_{l}\left(x\right)$ is made [5,55], extending the expansion up to l=30. The final length for these calculations is set to 70 Å, which allows one to deal appropriately with the long-range oscillations of h(r) about zero (for more details see [2,33]).

BHP Fourier space triplet calculations at SP4 minimize with respect to t(r) the functional ℑ[t(r)] given in Equation (12c). The initial $t_{0}\left(r\right)$ is taken as $t_{0}\left(r\right)=h\left(r\right)$, and the integration range of distances $\tilde{R_{max}}$ is set to: (a) 70 Å, and (b) 100 Å. Two *r*-distance discretizations are studied: Δr=0.01 Å and 0.005 Å, which in defining t(r) imply 7001 points in 0≤r/Å≤70 (with 0.01), or 20,001 points in 0≤r/Å≤100 (with 0.005). Concomitantly, Fourier t(k)-values are treated in the same way by taking in each case equivalent discretizations (e.g., 20,001 points in 0 ≤ *k*/Å^−1^ ≤ 100, with Δ*k* = 0.005 Å^−1^). The numerical method chosen is a combination of conjugate gradient and pure gradient descents [5,10,56], as explained in detail elsewhere [34,35]. Such combination drives in general the minimization further down when the conjugate gradients “run out of steam” [56]. By doing so, a (double) sequence ${\left\{t_{\tau }\left(r\right);\;t^{\left(\tau \right)}\left(k\right)\right\}}_{\tau =0,1,2,\ldots }$ is obtained. It is worth remarking that a usual criterion for convergence in this context [10] is defined in terms of a ratio between ℑ[t(r)] and a reference density derivative quantity, by requiring $ℑ\left[t\left(r\right)\right]\leq \epsilon ∥\frac{\partial c_{2}\left(r;\rho_{N}\right)}{\partial \rho_{N}}{∥}^{2}$ where $\epsilon \approx 10^{-\nu }.$ Once convergence is reached at a given step $\tau_{C}$, the final calculations of $c_{ET}^{\left(3\right)}\left(k_{1},k_{2}\right)$ [5] can be performed, thereby giving $S_{ET}^{\left(3\right)}\left(k_{1},k_{2}\right)$ as indicated in Equation (11).

As regards the isothermal derivative of the direct correlation function, $\frac{\partial c_{ET2}\left(r;\rho_{N}\right)}{\partial \rho_{N}}$, the results obtained in [38] give two possibilities for carrying out the numerical treatment, since five states at 4.2 K were OZ2-studied with BDH+BHw: $\rho_{N}\left({Å}^{-3}\right)=0.02286713\pm \Delta \rho_{N}$; $\Delta \rho_{N}$ = 0, 0.002, 0.004. There is the simple derivative estimate involving the two states adjacent to SP4 (“finer” Stirling), and also the more accurate estimate obtainable with Richardson’s extrapolation that involves the four states around SP4 [57]. To visualize the situation, by denoting $x=0.004\;{Å}^{-3}$, Stirling estimate is accurate up to terms of order $O\left({\left(\frac{x}{2}\right)}^{2}\right),$ while Richardson’s extrapolation is accurate up to terms $O\left(x^{4}\right)$. Consequently, for the sake of comparison, these two algorithms are employed in this work. Now, the significant ranges $R_{Z}$ for nonzero pair direct correlation functions [53,54] arising from the whole OZ2 treatment must be considered. As these OZ2 computations show, the significant $R_{Z}$-zeros of the different $c_{ET2}\left(r;\rho_{N};R_{Z}\right)$ do not coincide with one another, and to calculate the density derivatives the $c_{ET2}\left(r\right)$-data regions needed are padded with zero-values. This extension with zero-values is also applied to every $c_{ET2}\left(r;\rho_{N}\right)$ beyond its selected $R_{Z}$ up to the limit fixed for the variational calculation of t(r) $\left(\tilde{R_{max}}=70\;Å;\;100\;Å\right).$ The latter action is consistent with the initial choice $t_{0}\left(r\right)=h\left(r\right),$ thus allowing for a long-range nonzero behavior of t(r) to develop. The longest ${\{R}_{Z}-\mathrm{zeros}\}$ applications may be expected to perform better, in response to the wider radial $c_{ET2}$-behavior that they contain. However, the effect of this operation deserves closer inspection. Therefore, separate computations based on the Richardson extrapolation are carried out with the two sets $\left\{c_{ET2}\left(r;\rho_{N};\;R_{Z}\right)\right\}$ corresponding to the minimal $R_{Z}$-zeros (m: ${\left\{R_{Z}\right\}}_{m}$, in between 9.9 Å−11 Å) and the maximal $R_{Z}-\mathrm{zeros}$ (M: ${\left\{R_{Z}\right\}}_{M}$, in between 10.2 Å−14.1 Å) obtained at the four densities (see the Supplementary Material).

The current minimizations have square norms of the derivative of $c_{ET2}\left(r;\rho_{N}\right)$ at state point SP4 that are $\approx 5\times 10^{6}.$ Note that: (a) rapid convergences are achieved, e.g., $\tau_{C}=$500–700 iterations; and (b) final ℑ[t(r)] values are $\approx 10^{-2}-10^{-3},$ which make $\epsilon \approx 10^{-9}-10^{-10}.$ At the final stages of the different minimizations, the convergences in the auxiliary function t(r) yield typically $\|t_{\tau }\left(r\right)-t_{\tau +1}{\left(r\right)\|}^{2}≲10^{-9}.$ Fourier transforms are performed via Fourier sums over the discretizations mentioned above. The behaviors of the auxiliary functions t(r) and t(k) are consistent with significant applications of the Fourier transform: the two functions tend effectively to zero as both *r* and *k* increase. In fact, by focusing the attention on the basic quantity t(r) it is worth remarking that the onset of its quick decay shows up in the region defined by the zeros $\left\{R_{Z}\right\}$ employed. To illustrate this point, some representative results, once convergence is reached, are quoted. Thus, for the case ${\left(\tilde{R_{max}}=70\;Å;\;\Delta r=0.01\;Å\right)}_{RM},$ employing Richardson’s extrapolation (R) and the maximal $R_{Z}-\mathrm{zeros}$ (M), one finds: (a) t(r=0)≅−2.338, t(k=0)≅−843.3; (b) $t\left(r=14\right)≅3.7\times 10^{-5},\;t\left(k=14\right)≅-1.2\times 10^{-3};$ (c) $t\left(r=43.17\right)≅-1.7\times 10^{-8},\;t\left(k=43.17\right)≅7.2\times 10^{-5}$; and (d) $\left|t\left(67\leq r\leq 70\right)\right|≲10^{-10},$ $\left|t(67\leq k\leq 70\right|≲10^{-5}$. With increasing *r* and *k* both t(r) and t(k) show very small and damped oscillations about zero (see t(r) in the Supplementary Material). Thus, there appears a t(r)-nonzero tail for distances greater than the longest maximal-$R_{Z}$ that defines the density-derivative nonzero range $\left(r<R_{Z}\right)$, albeit the related features are rather small. The further enlargement to $\tilde{R_{max}}=100\;Å$ only brings about very slight changes in the t(r) absolute values, thereby producing final $S_{ET}^{\left(3\right)}\left(k_{1},k_{2}\right)$ results in close agreement with the former at $\tilde{R_{max}}=70\;Å\;$(see Section 4). Among these changes in t(r), two may be worth mentioning: (i) within the essential region of nonzero density derivatives the values remain stable up to four/five decimal places; and (ii) for both $\tilde{R_{max}}=70\;Å$ and 100 Å and, roughly speaking, for distances in intervals within the range 17.6≲r/Å≲22.3, one finds a relative increase in the |t(r)| values with respect to their decay trends $\left(\left|t\left(r\right)\right|∼10^{-4}\right)$. As stated above, these changes do not alter the physics of the results obtained, although the second item might be subjected to a closer numerical inspection in future work. The foregoing general features are maintained under the different integration conditions used for ℑ[t(r)].

## 4. Results

### 4.1. **r**-Space Triplet Correlation Functions

**Figure 1:**
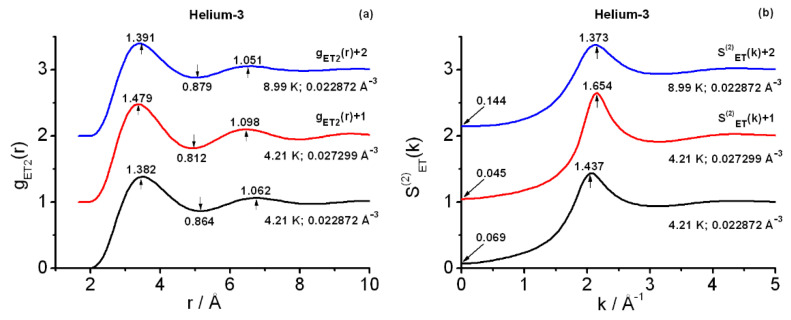
Pair instantaneous structures at state points $SP1\left(4.21\;K,0.0228717687\;{Å}^{-3}\right),\;\;SP2\left(4.21\;K,0.0272988971\;{Å}^{-3}\right),\;\;SP3\left(8.99K,0.0228717687\;{Å}^{-3}\right)$. (**a**) OZ2(BDH+BHw) radial correlation functions. (**b**) Associated OZ2(BDH+BHw) structure factors. Functions at SP2 and SP3 shifted by +1 and +2, respectively; marked are the actual salient features in each plot. OZ2 = pair level Ornstein–Zernike, BDH+BHw = Baxter–Dixon–Hutchinson procedure plus Baumketner–Hiwatari grand canonical corrections [44,45,46]. To avoid rounding-off errors in interpreting the actual values of the number densities employed in the calculations they are given in this caption up to ten decimal places.

Figure 1 contains the final results at the pair level for state points SP1, SP2, and SP3. One observes in Figure 1a the rightward shift and smoothing of the instantaneous radial structure $g_{ET2}\left(r\right)$ when the temperature is lowered (SP3-8.99 K vs. SP1-4.21 K) and the density is held fixed (0.022872 Å^−3^). Additionally, in Figure 1a the “compression” (or closer packing) of the instantaneous radial structure is apparent when the density is increased at constant temperature (4.21 K: SP1-$0.022872\;{Å}^{-3}\;\mathrm{vs}.$ SP2-$0.027299\;{Å}^{-3})$. These are the expected features of $g_{ET2}\left(r\right)$. In stark contrast with the results in the classical domain, the smoothing is consistent with the increasing quantum delocalization of the particles under diminishing temperatures. For visualization purposes, this general smoothing of structures is perhaps easier to grasp via the semiclassical Feynman-Hibbs Gaussian picture [13]. In this semiclassical approximation, each quantum particle is described by a thermal Gaussian packet. The pair radial function arises from a convolution, involving two of these packets, which smears out the sharper features of the pair radial function between the centers of the packets. With diminishing temperatures the width of the Gaussian packet increases, and through the convolution so does the smoothing effect (see [11] for further references and some related calculations).

The accuracy of the foregoing results for helium-3, as studied with SAPT2, can be assessed via the information in Figure 1b, where the pair instantaneous structure factors $S_{ET}^{\left(2\right)}k$ are shown. In this regard, key pieces are the values $S_{ET}^{\left(2\right)}k=0,$ or equivalently the corresponding isothermal compressibilities, which compare very well to the experimental values [48]. Thus, one finds: SP1 ($\chi_{T}/bar^{-1}=0.005715$ (experimental), 0.005202 (computed)); SP2 $(\chi_{T}/bar^{-1}=0.002383$ (experimental), 0.002844 (computed)); and SP3 $(\chi_{T}/bar^{-1}=$ 0.005003 (experimental), 0.005071 (computed)). The foregoing values are estimates obtained: (1) via three-point quadratic interpolation of the experimental (pressure, volume)−data; (2) by selecting one of the representative structure factors arising from the BDH+BHw iterative procedure (five BHw iterations, and the largest-$R_{Z}$ case) [38,53,54]. Although other estimates are possible, for the current purposes, their overall influence on the **r**- and **k**- results is slight. In relation to this, the averages over $R_{Z}$-results for the ET2-computed isothermal compressibilities are worth quoting: $\bar{\chi_{T}}/bar^{-1}=$ 0.005491 (SP1), 0.002867 (SP2), 0.005090 (SP3). Accordingly, no averages of structure functions over the significant $R_{Z}$-ranges [53,54] are carried out in this work when calculating $\frac{\partial c_{2}\left(r;\rho_{N}\right)}{\partial \rho_{N}}$. Even though OZ2 applications to the instantaneous $g_{ET2}\left(r\right)$ are approximations [11], not only these computations yield $\chi_{T}$-values close to the experimental values, but also keep their correct ordering. Given the high sensitivity of this thermodynamic quantity, the current structures obtained at the pair level are to be regarded as a very good representation of the actual helium-3 structures under the conditions studied. Their use as data input to triplet closure calculations is then fully reliable. Further support for the previous statement is provided by the PIMC calculation of pressures, which yields: SP1$(\frac{p}{bar}=$33.64 (experimental), 32.44 (computed)); SP2$(\frac{p}{bar}=$82.68 (experimental), 80.20 (computed)); SP3$(\frac{p}{bar}=$55.25 (experimental), 55.05 (computed)). (Error bars for the PIMC pressures remain below 2%).

For the sake of comparison, it is worthwhile to mention that, after rounding-off to three decimal places, the salient features of $g_{ET2}\left(r\right)$ at SP4 (4.2 K; 0.022867 ${Å}^{-3})$ as obtained in [38] coincide with those shown in Figure 1a for SP1 (see the Supplementary Material). Typical instantaneous estimates of the isothermal compressibility at SP4 were $\chi_{T}/bar^{-1}\approx 0.0062$ as ET2-computed [38], with the experimental estimate(s) being $\approx 0.00575\;bar^{-1}$ [48]. (As regards the pressure at SP4, PIMC gave $\frac{p}{bar}=$32.11 [38]). To grasp the SP1-SP4 differences in the ET2 computed $\chi_{T},$ one must remark the combined effect of the substantially larger $N_{S}$ (1372 vs. 1024) plus the more extensive statistics of the pair structures in the present work than what could be achieved in [38]. The current sampling yields highly accurate SP1 structures, hence it is the very low-*k* region of $S_{ET}^{\left(2\right)}$ (e.g., $\chi_{T}$-computation at k=0) which benefits greatly from this, whereas the rest of the structure factor remains practically invariable. The previous discussion lends additional quantitative support to the SP1-SP4 great proximity expected for their triplet structures.

**Figure 2:**
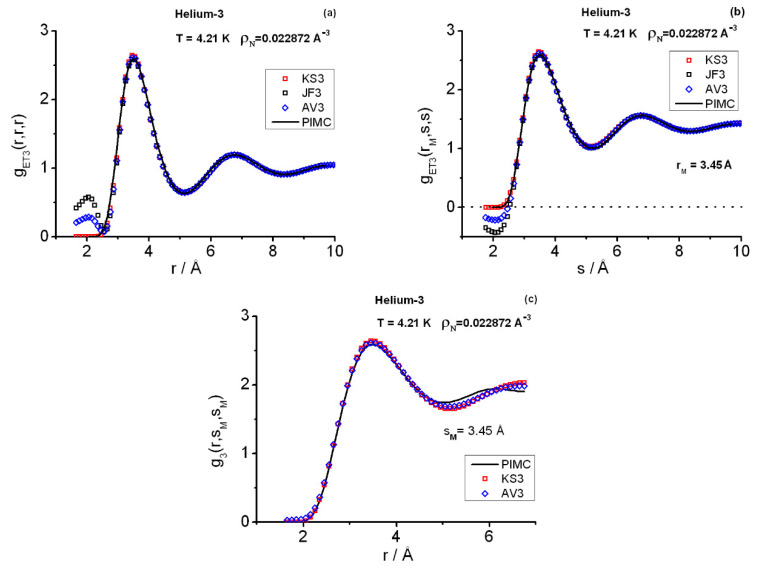
Triplet structures in **r**−space at state point SP1. (**a**) Equilateral correlations. (**b**) Isosceles correlations at the $r=r_{M}$ slice in the close vicinity of the position of the equilateral first peak. (**c**) *r* profile of the isosceles heights at the $s=s_{M}$ distance in the close vicinities of the positions of the recorded isosceles maxima. KS3 = Kirkwood superposition, JF3 = Jackson–Feenberg convolution, AV3 = (KS3 + JF3)/2, PIMC = path integral Monte Carlo.

**Figure 3:**
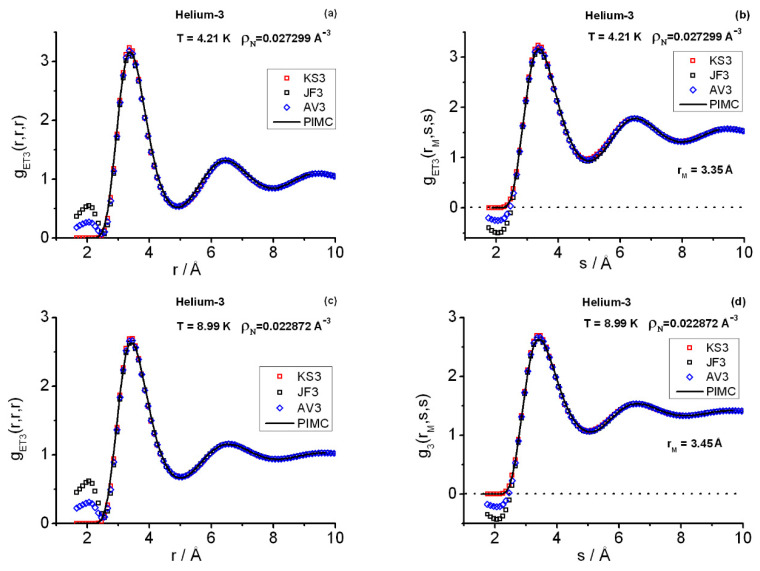
Triplet structures in **r**−space. (**a**) Equilateral correlations at state point SP2. (**b**) Isosceles correlations at SP2 at the $r=r_{M}$ slice in the close vicinity of the position of the equilateral first peak. (**c**) Equilateral correlations at state point SP3. (**d**) Isosceles correlations at SP3 at the $r=r_{M}$ slice in the close vicinity of the position of the equilateral first peak. The rest of the symbols as in Figure 2.

Figure 2 and Figure 3 display the triplet instantaneous correlations computed with PIMC and closures and, for definiteness, Table 1 contains the PIMC salient features. As regards the PIMC results, the equilateral correlations $g_{ET3}\left(r,r,r\right)$ follow closely the pattern set by the pair structures $g_{ET2}\left(r\right)$: (1) although the triplet salient features are more pronounced, the positions of their peaks and valleys at a given state point are close to those at the pair level (Figure 2a and Figure 3a,c); and (2) their relative heights are in correspondence with those at the pair level. In addition, their expected asymptotic behavior for long distances is that they tend to unity [33]. The isosceles correlations $g_{ET3}\left(r,s,s\right)$ are plotted at the *r*-slice for the equilateral main peak positions $r_{M}$ (Figure 2b and Figure 3b,d). A relevant isosceles trait is that for long *s*-distances these correlations do not tend to unity in general, but to the limiting pair value $g_{ET2}\left(r_{M}\right)$ [33]. Owing to the range of distances $\left(\frac{L}{4}\right)$ scanned in the simulations, the foregoing asymptotic features are only hinted at by the oscillations in the related graphs. Nevertheless, PIMC runs at very low densities and the current closure applications (see below) indicate that the calculations are correct and would show these behaviors if allowances for much longer *L* and computational time were made. As regards the closure applications, three main points that agree with previous experience are to be commented [6,36]. First, the JF3 (AV3) bad behavior for short-range distances. Second, the remarkably good fitting provided by the closures when considered globally. Third, the closure departure shown in Figure 2c from the exact PIMC behavior. This departure is apparent as one inspects longer *r*-distances in the isosceles correlations measured with $g_{ET3}\left(r,s_{M},s_{M}\right),$ where $s_{M}$ is a distance in the close vicinity of the main peak of $g_{ET3}\left(r,r,r\right)$, i.e., Figure 2c shows the heights in the proximity of the main peaks belonging to increasing-*r* slices. One also notices that the use of the enlarged pair structures, incorporating grand-canonical corrections for the calculations of the closure values, is consistent with the PIMC canonical triplet structures.

Once again, a combined use of the closures KS3, JF3, and AV3 yields surprising results [2,6,36]. One can observe the excellent overall fit they give of the PIMC results for distances ≳2.5Å. The short-range distance behavior is correctly described by KS3, although JF3 clearly fails. Closure AV3 reduces the magnitude of such JF3-failure, but it is not sufficient to fix it. In the cases analyzed here, a combination of KS3 for the short-range distances, say $r_{0}≲2.75Å,$ and AV3 for the distances beyond yields a very good representation of the triplet correlations in **r**-space (recall the in-built correctness of the asymptotic behavior of closures). The question of amending the closure failures remains intriguing and, because of the current applications to helium-3, may be more focused now. The instantaneous results in **r**-space obtained so far point decidedly to a prominent role played by the underlying pair correlations in shaping the triplet correlations when strong quantum diffraction effects are present. In relation to the quantum hierarchy of (instantaneous) correlation functions, we owe this theoretical pair-based picture to closures.

### 4.2. **k**-Space Triplet Structure Factors

**Figure 4:**
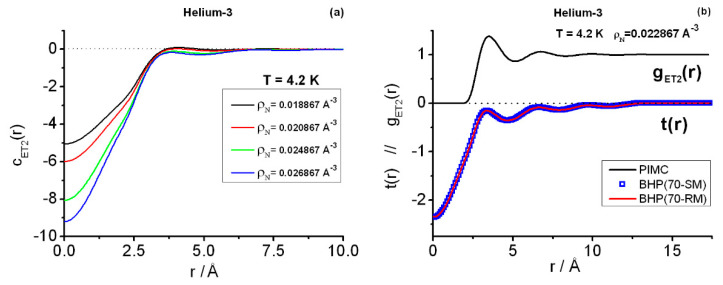
Pair instantaneous structural information relevant for the **k**-space triplet study at state point $\mathrm{SP}4\left(4.2\;K,\;0.02286713\;{Å}^{-3}\right)$ (**a**) Pair direct correlation functions $c_{ET2}\left(r\right)$ at the four states adjacent to SP4 along the 4.2 K isotherm [38]. (**b**) Pair radial correlation function (upper plot) [38] and BHP-closure t(r) functions obtained via two different numerical density-derivatives (Equations (12)). BHP = Barrat–Hansen–Pastore [5], $\left(\tilde{R_{max}}=70\;Å;\Delta r=0.01\;Å\right)$, utilizing the $c_{ET2}\left(r;R_{Z}\right)$ associated with their maximal (M) $R_{Z}$-values as arising from the BDH+BHw treatment. Using the state points in panel (**a**), the derivatives in panel (**b**) based on the maximal $R_{Z}$-zeros are denoted by: SM = Stirling derivative involving the two closest points to SP4, RM = Richardson extrapolation involving the four points.

Figure 4a displays the instantaneous direct correlation functions $c_{ET2}\left(r\right)$ associated with the instantaneous $g_{ET2}\left(r\right)$ at the four state points about the target state point selected for the triplet calculations in **k**-space, SP4 $\left(4.2\;K;0.022867\;{Å}^{-3}\right)$ [38]. It is interesting to note the fast decay to zero of these functions. Figure 4b contains representative results for the closure function t(r), Equation (12b), as arising from the variational Barrat–Hansen–Pastore (BHP) $\left(\tilde{R_{max}}\;=70\;Å;\Delta r=0.01Å\right)$ calculations. One observes that, apart from the deeper bowl within short range, t(r) maintains its oscillations somewhat close to those of the starting choice $t_{0}\left(r\right)=h_{ET2}\left(r\right)=g_{ET2}\left(r\right)-1$. The effect of the derivative computation $\frac{\partial c_{2}\left(r_{12};\rho_{N}\right)}{\partial \rho_{N}}$ on the final t(r) seems unimportant on the scale of the graph, as seen in the curves of the applications of Richardson’s extrapolation (RM: four-point derivative) and Stirling (SM: two-closest-point derivative). Other details such as the effects of varying the ranges $\left\{R_{Z}\right\}$ considered or using a larger $\tilde{R_{max}}$ with a finer discretization, e.g., $\left(\tilde{R_{max}}=100\;Å;\Delta r=0.005\;Å\right)$, will be addressed below.

**Figure 5:**
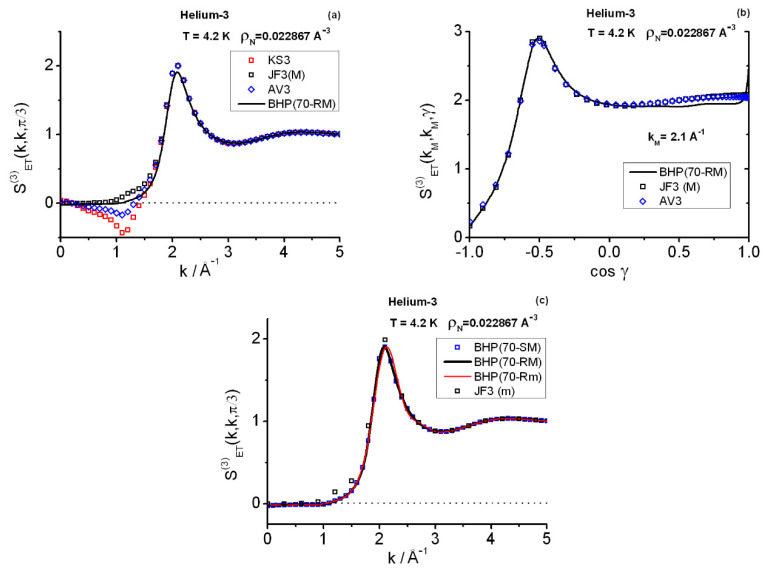
Triplet instantaneous structure factors at state point SP4. (**a**) Equilateral components. (**b**) Isosceles components in the close vicinity of the equilateral maximum at $k=k_{M}$. (**c**) Comparison of different applications using the sets $\left\{c_{ET2}\left(r;R_{Z}\right)\right\}$ corresponding to the maximal (M: 10.2 Å−14.1 Å)) and minimal (m: 9.9 Å−11 Å) $\left\{R_{Z}\right\}$-sets: BHP $\left(\tilde{R_{max}}=70\;Å;\Delta r=0.01\;Å\right)$ with Stirling (S) and Richardson’s extrapolation (R) derivatives, JF3 = Jackson–Feenberg. Continuous lines treated with splines.

In interpreting the **k**-space results, one must keep in mind that $S_{ET}^{\left(3\right)}\left(k_{1},k_{2},\gamma \right)$ is essentially the Fourier transform of the involved correlation function $H_{ET3}\left(r_{12},r_{13},r_{23}\right),$ of which $g_{ET3}\left(r_{12},r_{13},r_{23}\right)$ forms a distinctive part [11]. To draw significant conclusions from this line of thought, one would need an exhaustive $g_{ET3}$-knowledge that goes beyond the equilateral/isosceles computations [6,11]. Consequently, Figure 5 contains the results based on the alternative given by direct correlation functions. The panel (a) shows the equilateral instantaneous components $S_{ET}^{\left(3\right)}\left(k,k,\pi /3\right)$ obtained with the four closures: KS3, JF3, AV3, and BHP. One observes good agreement among the different approaches past the main peak $(k≳2.1\;{Å}^{-1}$), which is related to the decreasing influence of $c_{ET}^{\left(3\right)}$ (Equation (11)), i.e., JF3 dominates this behavior. The interesting region is $0\leq k\left({Å}^{-1}\right)≲2.1,$ where discrepancies among the closures are important within $0.5<k({Å}^{-1})≲1.5$. In connection with this region, one notes the following facts: (a) BHP estimates are based on Equation (12d) and show that $S_{ET}^{\left(3\right)}\left(k_{1}=0,k_{2}=0\right)<0,$ e.g., $S_{ET}^{\left(3\right)}\left(k_{1}=0,k_{2}=0\right)=-0.0213$ for ${\left(\tilde{R_{max}}=70\;Å;\Delta r=0.01\;Å\right)}_{RM}.$ Notice that Equations (12a) and (12d) are an integral part of BHP but not of the other three closures. (b) Actually, BHP yields (small) negative values for the equilateral $S_{ET}^{\left(3\right)}\left(k,k,\pi /3\right)$ components within $0<k\left({Å}^{-1}\right)≲1,$ e.g.,$\;S_{ET}^{\left(3\right)}\left(k_{1}=0.5,k_{2}=0.5,\frac{\pi }{3}\right)=-0.0112,\;\;S_{ET}^{\left(3\right)}\left(k_{1}=1,k_{2}=1,\frac{\pi }{3}\right)=-0.0045$. (c) In marked contrast, KS3 (and AV3) gives positive results at and near $(k_{1}=k_{2}=0$), although this trend changes quickly: negative results, larger than the BHP’s, arise for $0.3≲k\left({Å}^{-1}\right)≲1.4$. The intermediate AV3-character between KS3 and JF3 makes AV3 show a less pronounced behavior than KS3, but this is still far from BHP. To complete the interpretation of Figure 5a, some additional equilateral BHP components may be worth giving here: $S_{ET}^{\left(3\right)}\left(0.8,0.8,\frac{\pi }{3}\right)=-0.0109,$ $S_{ET}^{\left(3\right)}\left(1.5,1.5,\frac{\pi }{3}\right)=0.1575,\;S_{ET}^{\left(3\right)}\left(2.1,2.1,\frac{\pi }{3}\right)=1.9039,\;S_{ET}^{\left(3\right)}\left(2.7,2.7,\frac{\pi }{3}\right)=0.9814,$ $S_{ET}^{\left(3\right)}\left(3.1,3.1,\frac{\pi }{3}\right)=0.8744$.

In addition to the equilateral features, Figure 5b gives the isosceles components in the close vicinity $\left(k_{M}=2.1\;{Å}^{-1}\right)$ of the maximum of the equilateral components. Discrepancies between JF3 and AV3 are small (KS3 details can be inferred from the graph). BHP results follow very closely the JF3/AV3 trend within 1.32≲γ≤π, but they depart noticeably from it for 0≤γ≲1.32.

Figure 5c displays the very little global effect that using two sets of ranges of distances $\left\{R_{Z}\right\}$ has on the BHP triplet equilateral calculations. In this case, with the use of Richardson’s extrapolation, the ranges are in between: (Rm) 9.9 Å−11 Å for the minimal set ${\left\{R_{Z}\right\}}_{m}$, and (RM) 10.2 Å−14.1 Å for the maximal set ${\left\{R_{Z}\right\}}_{M}$. (As a reference, JF3 results arising from the set ${\left\{R_{Z}\right\}}_{m}$ are also shown). To complete this description some numerical values are worth quoting. For example, using $\left(\tilde{R_{max}}=70\;Å;\Delta r=0.01\;Å\right),$ the double-zero momentum transfer $S_{ET}^{\left(3\right)}\left(0,0\right)$ undergoes a relatively large change: (a) −0.0213 for Richardson-maximal (RM), (b) −0.0155 for Richardson-minimal (Rm), although the respective absolute values are certainly small and agree qualitatively. As regards the main peak heights arising from the latter applications, one finds for $S_{ET}^{\left(3\right)}\left(2.1,2.1,\frac{\pi }{3}\right)$ the values: (a) 1.9039 (RM), and (b) 1.8947 (Rm). Another issue shown in Figure 5c relates to the little overall importance of employing Stirling (70-SM) or Richardson (70-RM) derivatives in the current case, a fact that could change dramatically under conditions involving higher densities or more structured $\left\{c_{2}\left(r;R_{Z}\right)\right\}$ functions. In connection with this, as was to be expected, relatively appreciable effects occur for low-*k* wavenumbers, e.g., the double-zero momentum transfer for which 70-SM yields $S_{ET}^{\left(3\right)}\left(0,0\right)=-0.0275$. The discrepancies between 70-SM and 70-RM can affect the third decimal places as observed in the equilateral components, but the relative importance diminishes with increasing *k*-wavenumbers due to the BHP-properties of $S_{ET}^{\left(3\right)}\left(k,k,\frac{\pi }{3}\right)$ (e.g., it monotonically increases for 0.9≲k/Å≲2.1, remaining above 0.874 for larger wavenumbers).

At this point, one must also comment the effect of increasing the variational range of distances $\tilde{R_{max}}$ for obtaining t(r), together with the decrease in the *r*-discretization interval. In this regard, BHP calculations of $S_{ET}^{\left(3\right)}\left(k_{1},k_{2},\gamma \right)$ with ${\left(\tilde{R_{max}}=100\;Å;\Delta r=0.005\;Å\right)}_{RM}$, and their equivalent sizes in **k**-space, produce results perfectly consistent with those of the former choice ${\left(\tilde{R_{max}}=70\;Å;\Delta r=0.01\;Å\right)}_{RM}$: roughly speaking, one finds at least the first four or five decimal figures stable.

The lack of exact PIMC results at SP4 for $S_{ET}^{\left(3\right)}\left(k_{1},k_{2},\gamma \right)$ precludes a discussion of the relative merits of the closures employed in this work. However, some points allow one to make an educated guess. These points are: (a) the exactness of Equation (12d); (b) the diminishing influence of the triplet direct correlation function as wavenumbers *k* increase; and (c) the previous experience gained when studying liquid para-hydrogen [36]. On these grounds, it can be assumed that BHP gives a more acceptable description than the other three closures. Besides, pilot PIMC results at SP4 (2400 kpasses; $N_{S}\times P=128\times 66$) indicate that [58]: (d) an equilateral negative-region is expected to be a possible genuine feature of the triplet instantaneous structure factor (compare with [36]); and (e) there is a better behavior of BHP in such region, as deduced from the following partial median $S_{ET}^{\left(3\right)}\left(k,k,\frac{\pi }{3}\right)$-estimates obtained with PIMC: (i) at $k=0.5\;{Å}^{-1}$, median ≈−0.007, and (ii) at $k=1\;{Å}^{-1}$, median ≈−0.045. The latter magnitudes are not final and differ quantitatively from BHP’s, but they signal the negative trend. Accordingly, the pronounced negative region found in KS3 (and AV3) for the equilateral components is likely unphysical, which should be consistent with the discrepancies between PIMC and KS3 for the salient **r**-isosceles features shown (for SP1 ≈SP4) in Figure 2c. More relevant quantities, such as the actual intensities of the $S_{ET}^{\left(3\right)}$ main equilateral peak and its associated isosceles features, are also expected to be better captured by BHP, although only final PIMC results will settle this question. In relation to this, note that the individual $S_{ET}^{\left(3\right)}$-values fluctuate strongly when PIMC-sampling the configurations [36]. Therefore, the computation of precise average values entails very long run lengths in this type of calculations.

## 5. Conclusions

The current computational structure study has dealt with supercritical helium-3. Real space and Fourier space properties of the triplet instantaneous correlations have been investigated.

As regards the real space results, the exact PIMC equilateral and isosceles features show the influence of independent variations in temperature and in density. Thus, the structure is smoothed by the decrease in temperature, whereas it is sharpened by the increase in density. When inspecting the equilateral correlations, one observes that the salient features, i.e., positions and heights of the peaks and valleys, follow the patterns set by the pair correlations, albeit the equilateral triplets show far more pronounced first peaks and valleys. These traits are in accordance with what was obtained for the hard-sphere fluid [2] and liquid para-hydrogen [6].

From the comparison with PIMC one finds, once again, that the triplet closures used (KS3, JF3, AV3) reveal themselves as a great help in providing real-space physical pictures of triplet correlations in fluids with quantum behavior. This closure usefulness is thus consistent with that found in other applications [2,6]. One might expect this positive working of closures to occur when studying fluid helium (far from quantum exchange), because of its similarity to a quantum hard-sphere fluid [2,16]. Speculations are fine when they are consistent with known related facts, but there is nothing like direct proof. The current applications to helium-3 fill the conditions to be analyzed in that a continuous interparticle potential (as complementary to the hard-sphere potential) plus very strong quantum diffraction effects (as an extension to para-hydrogen conditions) are dealt with at the same time. The main conclusion is that pair correlations contribute decisively to shape the correlations at the triplet level. A combination of KS3 for small ranges of distances plus the use of the intermediate AV3 = (KS3 + JF3)/2 beyond these ranges yields a very good representation of the exact correlations. On the one hand, this (KS3+AV3)-representation is surprisingly accurate for: (1) the equilateral correlations over long ranges of distances, and (2) the isosceles correlations over short-medium ranges of distances. On the other hand, (KS3+AV3) loses predictive power for isosceles correlations beyond the ranges mentioned. Even though ranges of distances more general than those considered herein remain to be computed, the variety of quantum systems and conditions studied so far indicates that this usefulness of the closures utilized is not a fortunate coincidence. Therefore, it should be regarded as a general fact for fluids with strong quantum behavior. Although it is not fully clear how to continue expanding Equation (9), because of the convergence properties, this goal seems now to deserve a try.

The triplet instantaneous structure factor computations with closures at (T=4.2 K, $\rho_{N}=0.022867\;{Å}^{-3})$ show the important role played by the triplet direct correlation function. Because of Equation (11), beyond the region of the main peak $k≳2.1\;{Å}^{-1},$ the smallness of the latter function makes KS3, JF3, AV3, and BHP be in close agreement with one another on the equilateral component values, and also on the isosceles components for large angles $\gamma ≳75^{{}^{\circ}}.$ At $\gamma =60^{{}^{\circ}}$ BHP separates from the rest at the main peak, and far more noticeably within $0\leq k/{Å}^{-1}<1.5$. The same sort of discrepancies are found when inspecting the isosceles components, e.g., at $k=2.1\;{Å}^{-1},$ for angles $\gamma ≲75^{{}^{\circ}}.$ By construction JF3 takes positive values all over the possible ranges of wavevectors. However, KS3 (and AV3) and BHP take negative equilateral values for low-*k* wavenumbers. BHP (absolute) negative values are small, but this does not happen to KS3 which near $1\;{Å}^{-1},$ displays a pronounced dip below zero. Given the KS3 defect in reproducing the spatial triplet isosceles correlations as the ranges of distances are enlarged, its overall **k**-space behavior cannot be regarded as correct. The same may be said of AV3. BHP seems, however, better adapted to the task of giving better estimates of triplet structure factors. A reason for this is Equation (12b), an exact relationship which is a BHP’s in-built feature. The negative-valued region for the equilateral components obtained in the BHP calculations finds qualitative support in pilot PIMC results. Accordingly, one is led to surmise that such negative region should be a genuine triplet fact. Furthermore, the calculation of estimates of the double-zero momentum transfer component, which is a response property of a quantum fluid independent of the structural description [6], can be better carried out with the centroid correlations due to the exact OZ3 framework they provide.

There is ongoing work, involving PIMC and closures, which is focused mainly on the delicate problem of determining facts as accurately as possible about triplets in **k**-space for fluids with quantum behavior. The results, covering the instantaneous and the centroid structures, will be the subject of a future article.

## Figures and Tables

**Table 1:** Detail of the salient features of the PIMC triplet calculations in the canonical ensemble. The reported positions and correlation values are in the close vicinities of: fp = first peak, fv = first valley, sp = second peak, sv = second valley. Distance $r_{M}$ is in the vicinity of the position of the equilateral first peak. For notational convenience: $r=r_{12},s=r_{13},u=r_{23}.$ Statistical error bars in the $g_{ET3}$ values are ≤1%.

$g_{ET3}\left(r,s,u\right)$	SP1	SP2	SP3
	$4.21\;K;0.022872\;{Å}^{-3}$	$4.21\;K;0.027299\;{Å}^{-3}$	$8.99\;K;0.022872\;{Å}^{-3}$
Equilateral r=s=u $\left(r,g_{ET3}\right)$			
fp ($r_{M})$	(3.45, 2.588)	(3.35, 3.164)	(3.45,2.632)
fv	(5.15, 0.633)	(4.95, 0.522)	(5.05, 0.665)
sp	(6.75, 1.193)	(6.45, 1.316)	(6.55, 1.154)
sv	(8.35, 0.910)	(7.95, 0.846)	(8.15, 0.937)
Isosceles $r=r_{M};s=u$ $\left(s,g_{ET3}\right)$			
fp	(3.45,2.588)	(3.35,3.164)	(3.45,2.632)
fv	(5.25,1.008)	(4.95,0.933)	(5.05,1.055)
sp	(6.75, 1.560)	(6.45, 1.780)	(6.55, 1.537)
sv	(8.35, 1.294)	(7.95, 1.315)	(8.15, 1.332)

## Supplementary Materials

The following supporting information can be downloaded at www.mdpi.com/article/10.3390/e25020283/s1. r-space directory: final pair radial correlation functions at state points SP1 and SP4 (files: g2r_et_bdhbw.sp1, g2r_et_bdhbw.sp4), PIMC triplet correlations at state point SP1 (file: g3rss_et_pimc.sp1); k-space directory: pair direct correlation functions at state points about SP4 (files: cr2_etbdhbw.sp4-2, cr2_etbdhbw.sp4-1, cr2_etbdhbw.sp4+1, cr2_etbdhbw.sp4+2), pair structure factors at state points SP1 and SP4 (files: sk2_etbdhbw5.sp1, sk2_etbdhbw.sp4), BHP-closure auxiliary function at SP4 (file: tr_et_bhp70RM.sp4), BHP and JF3 triplet structure factors at SP4 (file: sk3_etbhp70RM.sp4).
